# Anti-NMDAR antibodies as a new piece in schizophrenia’s puzzle

**DOI:** 10.4155/fsoa-2017-0009

**Published:** 2017-03-03

**Authors:** Antonio L Teixeira, Natalia P Rocha, Xiang Zhang

**Affiliations:** 1 Neuropsychiatry Program, Department of Psychiatry & Behavioral Sciences, McGovern Medical School, University of Texas Health Science Center at Houston, 1941 East Road, Houston, TX, USA

**Keywords:** autoantibody, immunology, NMDA receptor, schizophrenia

Schizophrenia is a chronic psychiatric disorder marked by psychotic symptoms (i.e., hallucinations and delusions), behavioral changes (e.g., apathy, social withdrawal) and cognitive dysfunction (e.g., executive impairment) [1]. Affecting roughly 1% of the general population, schizophrenia exerts a significant socioeconomic burden due to its severity.

From an etiopathogenic perspective, schizophrenia can be conceptualized as the clinical outcome of a series of genetic and/or environmental factors impairing brain development. Accordingly, environmental factors influencing the early development of the CNS, such as maternal infection, maternal stress, nutritional deficiency, among others, would play a major role in schizophrenia development as indicated by epidemiological studies [2]. Interestingly, it has been proposed that a common link between maternal infection/stress and the late development of schizophrenia would be a pro-inflammatory immune response [3].

In the last decade, a great attention has been dedicated to the role played by the immune system in the pathophysiology of schizophrenia. Indeed, different approaches implicate immune dysfunction in schizophrenia: *post-mortem* studies in patients showing microglial activation and enhanced expression of inflammatory molecules in areas implicated in the genesis of schizophrenic symptoms [4]; PET studies reporting microglial activation *in vivo* [5]; consistent changes in the blood profile of cytokines and lymphocytes toward a pro-inflammatory response and/or immune activation [6–8]; genome-wide association studies reporting association between schizophrenia and genetic loci of the major histocompatibility complex and other immune-related genes [9]; and positive results of trials with anti-inflammatory-based strategies [10]. It is worth emphasizing that if a major role of the immune system is confirmed in the pathophysiology of schizophrenia, this may open new venues for therapeutics in an area in high need of novel interventions.

Recently, a new piece has been added to the complex puzzle of the immunology of schizophrenia. In the largest cross-sectional study of its kind, Lennox *et al.* [11] assessed a series of neuronal cell surface antibodies (anti-NMDAR, anti-LGI1, anti-CASPR2, anti-GABA_A_R and anti-AMPAR) in the serum of 228 young subjects (aged 14–35 years) presenting with a first-episode psychosis and 105 healthy controls. The antibodies were determined by live cell-based assays that, in contrast to other routine serological methods as ELISA and Western blot, allow identification of conformational epitopes. Twenty (9%) of the patients had one or more of the neuronal cell surface antibodies in comparison with four (4%) of the controls, a difference that did not reach statistical significance even when adjusting for potential confounding factors. Patients were followed up 6 months after initial evaluation, and no clinically significant difference emerged between those positive versus negative for autoantibodies. When analyzed separately, the frequency of NMDAR antibodies differed between patients and controls. While seven patients (3%) exhibited NMDAR antibodies, no control did (p = 0.0204).

Antibodies to antineuronal cell surface receptors were initially described in cases of autoimmune encephalitis [12]. Autoimmune encephalitis are rare clinical conditions marked by brain inflammation caused by autoimmune mechanisms, mainly antibody-mediated ones. Several autoantibodies have been implicated, conferring high clinical heterogeneity due to the great variability of clinical symptoms and target population.

Originally described in 2007 as a paraneoplastic syndrome [13], anti-NMDAR encephalitis is the commonest autoimmune encephalitis. While its real incidence is unknown, some reports indicate that it can be higher than initially suspected, accounting, for instance, for over 20% of noninfectious encephalitis [14]. Anti-NMDAR encephalitis affects predominantly young women – in which it is frequently associated with ovarian teratoma – and children. Psychiatric symptoms may vary, including psychotic and manic features. These symptoms are reported by almost all patients, being often the first ones, prompting the search for a psychiatrist. This form of presentation usually delays the diagnosis of anti-NMDAR encephalitis until the development of dysautonomia and/or neurological signs such as cognitive impairment (fluctuation in the alert level, disorientation, amnesia), seizures and involuntary movements (dystonia, orofacial dyskinesia). A great concern is the description of cases with isolated psychiatric syndromes, in other words, without neurological or dysautonomic signs, in approximately 5% of patients in the largest anti-NMDAR encephalitis cohort [15]. Cases of purely psychiatric presentations have been reported with other autoantibodies as well [12].

In this context, the current study of Lennox *et al.* [11] is of great relevance as it investigates a series of autoantibodies related to autoimmune encephalitis in the largest sample of young patients with first episode psychosis typically seen in psychiatric centers so far. Based on the finding that seven patients (3%), but no control, exhibited anti-NMDAR antibodies, and they could not be identified solely on clinical basis, the authors propose to screen all patients at their first episode psychosis for autoantibodies. The study has limitations that the authors duly recognized, such as that antibodies were tested in the serum, not in the CSF. While assessing CSF could grant higher specificity, it is now debatable if this would determine better sensitivity [16]. The anti-NMDAR antibody-positive patients did not receive any immune-based treatment (e.g., plasmapheresis, immunoglobulin) as it has been proposed for autoimmune encephalitis. Even receiving standard psychiatric treatment, they have similar outcome than antibody-negative patients on the 6-month follow-up, casting doubt on the clinical meaning of the identification of anti-NMDAR antibodies in these group of subjects. Moreover, regarding the complex method used for assessing these autoantibodies, much work still has to be done to standardize the assays and to make them available and affordable. Accordingly, it is still premature to argue for screening all first-episode psychosis subjects for autoantibodies. Time will tell whether this is another not (or very limited) clinically meaningful ‘fashion’ involving autoantibodies, as happened with antibasal ganglia antibodies and obsessive–compulsive spectrum disorders in the late 1990s and early 2000s [17], or indeed has clinical implication as described for anti-aquaporin antibodies and neuromyelitis optica in recent years [18].

Probably the most important lessons from this and other studies [19] addressing anti-NMDAR antibodies in schizophrenia are: immune mechanisms may be directly involved in the pathogenesis of some cases of psychosis, and this suggests that the hypofunction of NMDA receptors in brain circuits could be one of the underlying mechanisms of schizophrenia. It is worth mentioning that anti-NMDAR antibodies bind to the GluN1 (NR1) subunit of the NMDAR, determining its internalization and, therefore, reducing its availability [20]. Either way these two pathways must be better explored in order to foster the development of biomarkers and new therapeutic approaches for schizophrenia.
